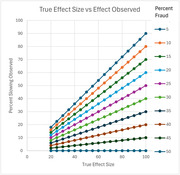# Improving the Chance of Success for Alzheimer’s Clinical Trials by Identifying and Mitigating the Risk of Fraudulent Practices

**DOI:** 10.1002/alz.095776

**Published:** 2025-01-09

**Authors:** Suzanne B. Hendrix, Patrick P O'Keefe, Jeffery Zhang, Kent Hendrix, Lixia Wang, Samuel P. Dickson

**Affiliations:** ^1^ Pentara Corporation, Salt Lake City, UT USA; ^2^ Drome Burgundy, San Francisco, CA USA

## Abstract

**Background:**

Companies recently identified clinical study sites engaging in fraudulent practices, reducing success in Alzheimer’s disease (AD) clinical trials. Successful trials are reliable despite possible victimization by these practices because these sites introduce a negative bias. Ease of simulating Alzheimer’s diagnosis documentation (based on clinical or blood markers) and clinical outcome data, coupled with inadequate oversight by Contract Research Organizations (CROs), facilitates deceptive practices.

Fraudulent practices at sites and with patients (“professional patients”) often go hand‐in‐hand and flourished during COVID. These must be aggressively addressed in the post‐COVID environment. Requiring and scrutinizing PET scans increases the chance of detecting fraud. Alarmingly, sites most prone to deceitful practices include predominantly underrepresented populations, thwarting inclusion efforts.

**Method:**

We reviewed completed and ongoing studies to estimate the percentage of potentially fraudulent sites. We developed medical audit methods to identify issues invisible in regulatory audits. Traditional data management and statistical analysis methods fail to identify these issues. We use expected enrollment and true effect size to estimate potential bias and added variability introduced by these sites. We propose methods for detecting fraud based on clinical and operational data.

**Result:**

In studies not requiring PET scans, approximately 10% of sites may be fraudulent, corresponding to a higher proportion of enrolled subjects (∼20‐30%). These sites can reduce observed effect size by 40% (estimated bias) at best and can completely cancel out a true treatment effect at worst. We estimate variability increases by 35‐100%, reducing our ability to observe significance. Algorithms to identify fraudulent sites will be gladly shared confidentially, but not broadly, to prevent sites from circumventing our algorithms.

**Conclusion:**

AD clinical trials affected by even a minority of fraudulent sites may have underestimated treatment effects. We developed a toolbox for combating these issues. It includes best practices for enhanced CRO oversight, incorporation of medical expertise in audits, and identification of problems based on targeted, blinded statistical algorithms. We freely share it with qualified researchers. Awareness of and taking an aggressive stance as a community against these fraudulent practices can safeguard AD research integrity, protect and benefit vulnerable populations, and accelerate development of effective therapeutics.